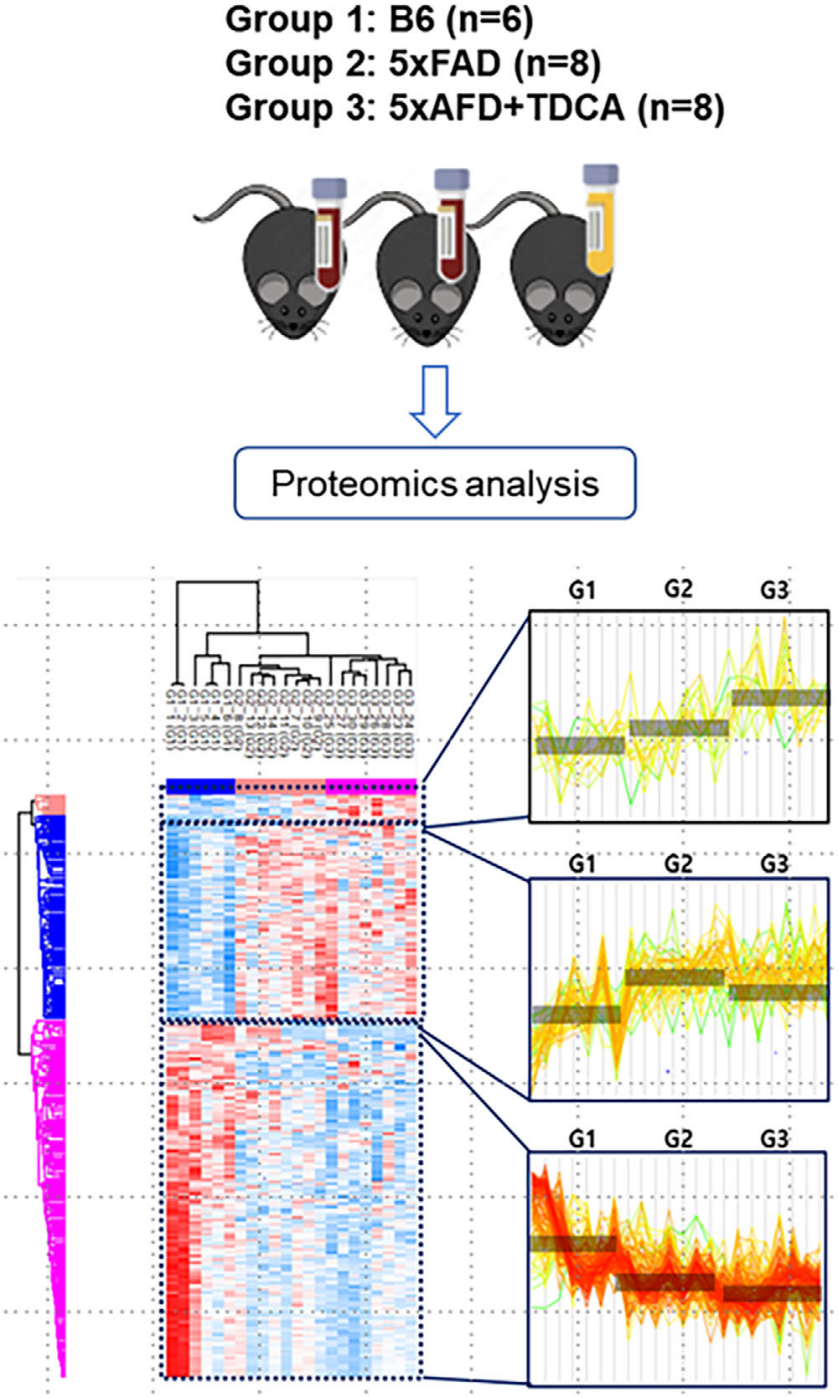# Evaluation of the therapeutic potential of nucerin, an oral TGR5 agonist HY209, in a murine model of alzheimer’s disease

**DOI:** 10.1002/alz.086687

**Published:** 2025-01-09

**Authors:** Siwon Kim, Jahirul Islam, Juyong Kim, Youngjae Koh, Jungjoong Hwang, Hyoung Tae Kim, Seung‐Yong Seong

**Affiliations:** ^1^ Shaperon Inc., Seoul Korea, Republic of (South); ^2^ Wide River Institute of Immunology, Seoul National University College of Medicine, Hong‐cheon Korea, Republic of (South); ^3^ Professor, Wide River Institute of Immunology, Seoul National University College of Medicine, Seoul Korea, Republic of (South)

## Abstract

**Background:**

Previously, we demonstrated therapeutic benefits following intraperitoneal delivery of the TGR5 agonist HY209 in 5xFAD, a transgenic mouse model of Alzheimer’s Disease (AD). Given the desirability of a more acceptable administration route for prolonged AD treatment, we assessed the efficacy of HY209 via oral delivery. This study aims to elucidate the therapeutic potential of NuCerin, an oral formulation of HY209, in the aforementioned AD model, while simultaneously identifying potential blood biomarkers indicative of NuCerin’s therapeutic action.

**Method:**

In both non‐Tg and 5xFAD mouse models, NuCerin (or its vehicle control) was orally administered for a duration of 12 weeks (30 mg/kg, b.i.d.). Subsequent cognitive assessment was undertaken using the Morris Water Maze (MWM) and Novel Object Recognition (NOR) tests. Amyloid plaque deposition in the cortical regions was measured after Thioflavin‐S staining. NeuN immunostaining was conducted to quantify mature neuronal population. LC‐MS/MS was employed on serum samples to identify potential protein biomarkers indicative of NuCerin’s efficacy.

**Result:**

The MWM and NOR tests indicated that oral delivery of NuCerin ameliorated cognitive impairment observed in 5xFAD murine models. Furthermore, a marked reduction in amyloid plaque deposition was observed in the NuCerin‐treated 5xFAD group. There was also an attenuation in the decline of NeuN‐positive neuronal populations in the NuCerin‐treated 5xFAD group as compared to the vehicle‐treated counterparts. Proteomic assessment highlighted an elevation in biomarker H levels in 5xFAD mice relative to non‐Tg mice; this increase, however, was mitigated in the NuCerin‐treated 5xFAD group.

**Conclusion:**

Our findings indicate that oral delivery of NuCerin attenuates the progression of Alzheimer’s disease, positioning NuCerin as a potential oral therapeutic for AD. Concurrently, we identified biomarker H as a potential biomarker to gauge NuCerin’s efficacy